# Well-Being of Lesbian, Gay, Bisexual Youth: The Influence of Rural and Urban Contexts on the Process of Building Identity and Disclosure

**DOI:** 10.3389/fpsyg.2021.787211

**Published:** 2022-01-13

**Authors:** Barbara Agueli, Giovanna Celardo, Ciro Esposito, Caterina Arcidiacono, Fortuna Procentese, Agostino Carbone, Immacolata Di Napoli

**Affiliations:** ^1^Department of Humanities, University of Naples Federico II, Naples, Italy; ^2^Department of Social and Developmental Psychology, Faculty of Medicine and Psychology, Sapienza University of Rome, Rome, Italy

**Keywords:** LGB, youth, well-being, identity construction, citizenship

## Abstract

The study investigates how the territorial community can influence the individual and social well-being of lesbian, gay, bisexual (LGB) youth and especially the recognition of their feelings and the construction of their own identity as well as their needs to be socially recognized. This research focuses on the experiences of 30 LGB individuals (23 males and 7 females), with a mean age of 25.07 years (*SD* = 4,578), living in urban and rural areas of Southern Italy. Focalized open interviews were conducted, and the Grounded Theory Methodology, supported by the Atlas.ti 8.0 software, was used for data analysis. The textual material was first coded, and then codes were grouped into five macro-categories: Freedom of identity expression in the urban and rural context, identity construction and acceptance process, need of aggregation and identification with the LGB community, role of the interpersonal relationship in the process of identity acceptance, socio-cultural context, and LGB psychological well-being. The results showed a condition common to the two contexts that we can define as “ghettoization.” The young LGB is alone in the rural area due to a lack of places and people to identify with and greater social isolation. On the contrary, although there are more opportunities in the urban area, young people feel stigmatized and ghettoized because “their places” are frequented exclusively by the lesbian, gay, bisexual, transexual, queer (LGBTQ) community. The work will extensively discuss the limitations of the research, future proposals, and the practical implications of the results.

## Introduction

The seminal work of D’Augelli (1994) opened the scientific debate on sexual orientation issues by introducing significant elements to understand the effects of gender-based stereotypes and social exclusion. Consequently, hetero-sexism was specifically depicted as a form of oppression, and further, in this respect, Harper (2005) highlighted the need to listen to the changing voices of the lesbian, gay, bisexual, transexual, queer (LGBTQ) people in the pursuit of liberation and well-being.

The LGBTQ experience has been strongly connected to cultural, social, and legal aspects of a given area. In many countries in the last half-century (1969–2019) the engagement in the Liberation Movement for LGBTQ citizenship has pursued *the imperative of leaving no one behind*, achieving basic goals in human rights recognition (Arcidiacono and Carbone, 2021). Therefore, the disclosure of non-binary sexual orientation and gender identity became a milestone in the life of LGBTQ youth. However, this experience had different perspectives in different contexts and social conditions.

Hence *heterosexism was “defined as an ideological system that denies, denigrates, and stigmatizes any non-heterosexual form of behavior, identity, relationship, or community*” (Herek, 1990, p. 316). Similar to racism and sexism, heterosexism manifested itself in two forms: cultural heterosexism (societal custom and institution) and psychological heterosexism (individual attitudes and behavior) (Herek, 1990). Therefore. LGBTQ people developed negative feelings toward their sexual identities as a consequence of living in a heterosexist society (Amodeo et al., 2018; Bochicchio et al., 2019).

Indeed, exposure to heterosexism, homosexual-related discrimination, perceived stigma, or other stressors were associated with poorer mental health for sexual minorities.

The minority stress theory (Meyer, 2003) postulated that sexual minorities experience unique stressors related to their sexual minority identity and negatively impact their health (Scandurra et al., 2020c,^2021^). Particularly, the minority stress theory distinguishes two types of stressors: distal stressors, which are external, such as prejudice or discriminatory events, and proximal stressors, which are internal stressors, negative internalized self-experiences such as internalized homophobia or transphobia, and psychological distress (Meyer, 2003).

The acquirement of integral identity had effects on individual and social well-being. Lingiardi and Baiocco (2015) described two connected processes: the building up and the integration of identity. The first one refers to the development of awareness about one’s sexual orientation. Meanwhile, the second one refers to accepting one’s own identity, resolving internalized homophobia, and communicating one’s sexual orientation to others.

Several scholars have already highlighted the importance of adopting an ecological approach in understanding the processes that explain the construction of identity and sexual orientations (Alderson, 2003; Shilo and Savaya, 2011). The ecological approach considers the close connection of internal individual factors as well as the interpersonal and territorial factors involved; the ecological model also facilitates the identification of the factors on which to intervene to improve the conditions of individual well-being as well as the identity processes (Prilleltensky et al., 2001). Evans and Prilleltensky (2007) added that to eliminate any form of oppression and limitation of well-being it is necessary to promote the experience of self-efficacy and self-esteem at an individual level; respect and affirmation at a relational level; and acceptance of diversity and solidarity at the community level.

In a study with gay individuals, Fingerhut et al. (2010) argued that those who were higher in gay identity expressions reported higher levels of psychological well-being. Still, at the same time, gay identity was significantly associated with exposure to both the distal stressor of discrimination and the proximal stressor of perceived stigma. Therefore, LGBTQ individuals show higher levels of psychological distress and lower levels of well-being (Everett, 2015; Watson et al., 2018), that is, social isolation and connectedness affect the well-being of LGBTQ youth (Scandurra et al., 2017; Garcia et al., 2020).

However, as a matter of fact, around the world, there are still specifically emerging challenges.

Connection with the LGBTQ community is an essential factor for the well-being of sexual minorities as it can buffer the effects of stigma and oppression that lead to minority stress (Meyer, 2003; Frost and Meyer, 2012). In this sense, it is essential to recognize the impact of prejudices during identity development and subsequent identification with the minority group (Everett, 2015; Scroggs and Vennum, 2020). For this reason, the LGBTQ community represents a protective factor toward distal and proximal stressors.

The social support of the LGBTQ community is significant as being part of an internal group that understands this specific stressor is a resilience factor (Fingerhut et al., 2010).

In this vein, the social support of LGBTQ depends on the physical and socio-cultural characteristics of the contexts to which the sexual minority belongs.

Contextual variables can affect coming out. Even today, in some contexts, it is common to adopt the principle that Boulden (2001) defined “*don’t ask, don’t tell*,” an approach whereby, if someone suspects a person’s homosexuality, they do not ask directly. In case he/she knows for sure, the homosexual person cannot explicitly share information about it. This modality, therefore, translates into the need to keep one’s sexual orientation secret and, therefore, negatively affects the acceptance process and the subsequent coming out.

This particular way of approaching homosexuality is an example of how hetero-sexism (Herek, 2004) is reflected in the behavior of communities and therefore contributes to the perpetuation of closure toward sexual minorities and discourages people from talking about it, feeding instead, the need to remain invisible.

The traditional values of which contexts are bearers and defenders are very often based on religious beliefs that have historically condemned homosexual conduct and contributed to the perpetuation of homophobia. Indeed, religious fundamentalism is closely related to homophobic prejudice. Hence different studies have shown that the basis of negative attitudes toward gays and lesbians is precisely the system of religious beliefs: many religious denominations deny homosexuality and therefore the faithful internalize these precepts and take a negative view of homosexuality (Whitley, 2009).

In these contexts, young people are more likely to experience social isolation and exasperated feelings of loneliness due to homophobia and pressure to adhere to heteronormative expectations (Hubach et al., 2019).

This aspect is one of the reasons that very often pushes young LGBTQ people to move away from their families, from their communities, and move to urban areas where they are more likely to find that support or, more generally, that lifestyle they seek.

In the city, it is more likely to find gay communities that encourage encounters; big cities offer more significant opportunities for socialization, anonymity, and sexual partners (Hubbard, 2012; Procentese and Gatti, 2020). Many studies highlighted adverse reactions from parents (D’Augelli et al., 1998; Willoughby et al., 2006; Baiocco et al., 2015) or siblings (Hilton and Szymanski, 2014; Pistella et al., 2020) and family rejection is investigated as a factor that could threaten the psychophysical well-being of young LGBTQ people at an individual level (Newcomb et al., 2019; Scandurra et al., 2020a,b).

Therefore, the need to leave their families and relocate meets their need to strengthen their acceptance, find support for their identity, and form social support networks. In the cities far from home, the first sexual experiences and the first step of accepting one’s sexuality occur (Annes and Redlin, 2012).

At first, in most studies, attention has mainly focused on considering the areas where sexual minorities are settling that is, in big cities, in contexts where the homosexual liberation movement had created the conditions for the settlement of the first LGBTQ communities and neighborhoods. Indeed, cities are the only possible contexts of life for sexual minorities because they are “*where the modern gay identity is constructed*” (Langarita Adiego, 2020, p. 1349).

Moreover, many studies highlighted the need to investigate rural and small town’ sexual minorities because they face more significant difficulties due to intense stigmatization (Swank et al., 2012), social exclusion (Waldo et al., 1998), and less access to support services (Fisher et al., 2014a,b). Moreover, they appear most disadvantaged in mental health, social service, and health care (Whitehead et al., 2016).

Furthermore, literature has amply demonstrated that sexual minorities living in rural areas are more likely to experience victimization and discrimination, less identification and engagement with the LGBTQ community, and less social support received (D’Augelli, 2006; Rickard and Yancey, 2018).

However, although many studies have focused on urban areas or highlighted the difficult living conditions experienced in rural contexts, different studies show that rural areas are not all anti-LGBTQ. Recent work has shown that LGBTQ individuals are out and accepted in rural areas; indeed, gay and lesbian identities in rural areas are not all closed, hidden, and oppressed (Kazyak, 2011).

As highlighted by Kazyak (2016), life experiences in rural areas are different and many people may experience certain conditions such as a slower pace of life or close relationships with family and friends as positive elements that allow them to face isolation and lack of connection with an LGBTQ community.

Compared to the rest of the European Union, Italy represents a particular case in terms of LGBTQ issues.

Callahan and Loscocco (2021) underline how socio-historical factors and social institutions, such as the Catholic Church, the family, and the political system, are among the main causes of resistance to the inclusion and legitimation of sexual minorities in Italy.

The presence of the Vatican on Italian territory represents a deterrent to the possibility of openness toward sexual minorities as well as an obstacle to laws that guarantee the rights of homosexual couples. Often in Italy, the laws that affect morality have gone through negotiations with the Vatican (Moscati, 2010).

Moreover, in Italy, according to the most recent FRA report (European Union Agency For Fundamental Rights [FRA], 2019), the LGBT community claims that its living conditions have worsened due to an increase of prejudice and intolerance and a lack of confidence in the real commitment of public institutions.

On this basis, the present research aimed to analyze, in an ecological approach, the acceptance and construction of LGB identity in Italian young people and their well-being, taking into particular consideration the influence of contextual factors, the territorial community of belonging. Indeed, the main purpose of the study was to highlight the conditions of discrimination perceived by young LGB people in their urban and rural contexts and how these conditions determine their perception of well-being. Moreover, the study is the first at an Italian level that considers the role of rural contexts for the well-being of LGB people.

## Materials and Methods

### Participants

The participants were 30 young LGB people (23 males and 7 females), aged 18–35 with a mean age of 25.07 years (*SD* = 4,578), living in metropolitan and rural areas of southern Italy. Specifically, 15 come from the metropolitan city of Naples (Campania), the third city in Italy by population and one of the most densely populated urban areas in Europe, and 15 from the province of Foggia (Puglia), a large, geographically extensive agricultural province, characterized by numerous municipalities with a very low population density.

Mainly, LGBTQ associations are territorially rooted in Naples, and the city is increasingly becoming a gay-friendly city. The LGBTQ community can find different reference points to have fun, discuss and participate in cultural events in clubs, bars, squares, and even beaches. In addition, there is the *House of Cultures and Reception* for LGBTQ people in Naples, the country’s first emergency municipal residence for LGBTQ people who are victims of discrimination or social marginalization. The situation in Foggia is different. Although many associations deal with the rights of sexual minorities and contrast all forms of discrimination, they have a shorter tradition than the Neapolitan realities.

Moreover, there are only a few gay clubs in the city and province and few places of aggregation and meeting.

The interviewees were recruited with a snowball or nominated sampling (Morse, 2010), considering sexual orientation, place of origin, and age range (young and young adults). This type of sampling involves choosing a certain number of individuals with specific characteristics in line with the research questions and asking them for other names to be interviewed. It is mainly used in cases where the population is made up of people who tend to hide their identity or are challenging to find, as in this case (Sullivan and Losberg, 2003). The group of participants was built and then extended using the social network of the participants themselves and the researchers.

For all participants’ characteristics (see Table 1).

**TABLE 1:** Participants’ characteristics.

Age	*M* = 25.07	*SD* = 4.578
***% N Sex***		
Male	76.7	23
Female	23.3	7
***Sexual orientation***		
Gay	70.0	21
Lesbian	16.7	5
Bisexual	13.3	4
***Context of belonging***		
Rural (foggia countryside)	50	15
Urban (naples)	50	15
***Marital status***		
Single	16	53.3
With a partner	14	46.7
***Profession***		
Student	56.7	17
Worker	40.0	12
Unemployed	3.3	1
***Religion***		
Practicing catholic	30.0	9
Non-practicing catholic	13.3	4
Atheist	53.3	16
Agnostic	3.3	1
*Total*	100	30

### Methods and Procedures

Data were collected through focalized open interviews (Legewie, 2006; Arcidiacono, 2016). This type of interview is based on a dialogical approach, that in the interaction requires some specific criteria: (a) to let the flow of the interviewee’s thoughts be as free as possible; (b) to deepen the meanings attributed to the investigation topic; (c) to consider the materials that emerge; (d) to explore personal and intimate aspects depicting the experience.

It gave interviewees enough freedom to share their life stories while narrowing down the narrative to specific areas. In this research, the grid of interviews included the following sites to explore: coming out; interpersonal relationships (families, friends, and partners); relationship with the context; discrimination and bullying; future; needs provided by services.

Participants were contacted by telephone, making an appointment with each of them, according to the most convenient days and places. In some cases, the participants, in turn, brought with them a friend to interview; the contact, in this case, did not take place directly.

The interviews took place at the interviewees’ home or, in some cases, at the interviewer’s home and lasted on average between 45 and 90 min.

After establishing the first contact with participants, informed consent was presented to them, explaining the research objective in question, the data disclosure methods, respect for anonymity, and the privacy regulations. In the case of an agreed agreement, the interview was carried out. The university ethics committee approved the entire procedure (CERP n. 18/2019 del 13/5/2019).

### Data Analysis

The interviews collected were transcribed *verbatim*, subsequently combined into a single corpus. Then, textual material was analyzed using the Grounded Theory Methodology (GTM) (Corbin and Strauss, 2008; Bryant, 2017; Charmaz and Belgrave, 2018), supported by the ATLAS.ti 8.0 software (Muhr, 2017).

The data analysis, carried out through a bottom-up approach, involved three coding phases. The first phase (open coding) started with the attribution of code to significant words and sentences; in the second phase (axial coding), the number of codes was reduced through a criterion of similarity of meaning in codes groups and framed in wider macro-categories. Finally, the third stage (selective coding), involved the abstract conceptualization of the data and the identification of the core category around which to articulate the complex interpretative model of the phenomenon (Corbin and Strauss, 2008).

The whole research was conducted by a team of senior and junior researchers, also composed of researchers belonging to the two different contexts. After a first reading of the textual material, the whole research team discussed the codes attributed to the text (open coding), identifying common and specific conceptual categories. The coding was then re-discussed with members of the local LGB community. The research steps and phases were following the guideline for qualitative research (Critical Appraisal Skills Programme, 2018).

## Results

The analysis of the textual material resulted in 130 codes, subsequently grouped into 12 categories and five macro-categories: Freedom of identity expression in the urban and rural context, identity construction and acceptance process, need for aggregation and identification with the LGB community, Role of the interpersonal relationship in the process of identity acceptance, socio-cultural context and LGB psychological well-being.

### Freedom of Identity Expression in the Urban and Rural Context

This macro-category collects categories that describe the peculiarities, the physical and socio-cultural characteristics of the contexts of belonging, especially the differences between urban and rural contexts.

In urban contexts, there seems to be greater freedom to say: “*there is an objective difference because in the city you create your own space, you have the opportunity of being able to create it, […]. In the village you are forced: to go out with people of your age, your school friends, or you are forced to do certain things that are good for the village: you have to get married, you have to consider being a man, so there are different laws*” (Interviewee 9, M, 25, rural); conversely, in rural contexts, the possibility of being discovered is more outstanding: “*in the small village there is the fear that everything can get caught and judged respectfully, as opposed to the big city where you can also find out about things, but still allow you to live more freely*” (Interviewee 7, M, 33, rural). Furthermore, in the countryside, the possibility of being the object of prying eyes is more likely: “*I realize that maybe if you walk hand in hand with your boyfriend and walk around the city, they may not give you as many strange looks as they do in the country*” (Interviewee 28, M, 19, urban).

The differences are due to a particular socio-cultural condition that characterizes the rural contexts, in which emerge closure and intense pressures to conformism: “*my village is the classic of the south, very closed, very retrograde, very backward, […] due to a cultural background of the people, of their own life*” (Interviewee 4, M, 25, rural).

However, the freedom of expression that characterizes urban contexts does not represent a large city’s whole area. In fact, in the case of Naples, there is a distinction between the historic center and the suburbs, since the historic center represents the most active part, with a more significant presence of young people and therefore the heart of social life: “*in the historic center, since there are many people of our age, one does not make any problems, places where perhaps there is more presence of elderly people I am also sorry to say that the presence of a political right party, however, could cause you a problem also for your physical safety, because it is not the first time that a person is also beaten for a public manifestation of too much affection*” (Interviewee 18, F, 20, urban).

A dimension that has a strong influence in both contexts is religion. Indeed, participants refer to a level of homophobia, which is legitimized by the Christian creed, which sees homosexuality as a perversion: “*bigotry yes, although perhaps things are changing, because in the end there are old generations of people who are truly bigots, and there is always discrimination, you are always afraid of the different person who is not like you; regardless of religion there will always be someone who will not think so.*” (Interviewee 22, M, 20, urban).

Finally, university marks a fundamental transition, from high schools, which are a closed and protected ecosystem, to an ecosystem open to exchange. The transition from high school to university is perceived as a positive event, as participants can interface with different peers, without fear of being stigmatized and can be freely themselves: “*I enrolled in university, which for me was a moment of strong social knowledge […] I interfaced with anyone, and dynamics were also born where I had my dynamic; my dynamic was of an unreported homosexual type, but simply because I told my friend to my colleague how beautiful this professor is, I never had to justify anything*” (Interviewee 29, M, 29, urban). Furthermore, the transition to university often involves the transfer from the rural context to the urban one: “*The fact of enrolling at university and then moving to a big city, to be able to have independence from my mother, helped me a lot. […] The environment of a big city or, in any case, being distant from your mother telling you what to do, what not to do, or where to go helped me to come out*” (Interviewee 4, M, 25, rural).

### Identity Construction and Acceptance Process

This macro-category includes categories that refer to the identity construction process, the experiences of the young people interviewed, and their needs and prospects. The method of building one’s identity and the consequent unveiling of one’s sexual orientation turned out to be the result of a series of intrapsychic and social factors and coming out represents a critical moment that is the last step in the acceptance process.

In this process, basic feelings are common in urban and rural contexts. In fact, in both contexts, feelings of diversity, inadequacy, and non-acceptance of one’s sexual orientation emerge. Participants assert that their path of awareness was influenced mainly by the diversity and non-acceptance experienced in the peer groups “*ten years ago there were no associations, there was no one to help, and you had no guidelines*” (Interviewee 10, M, 25, rural), “*there were no tools to identify it, and if I, allowed myself to say something like that, that is, that I was attracted to boys rather than girls, I was seen differently. So, I kept it to myself.*” (Interviewee 15, M, 25, rural).

It is difficult for young LGB to accept that their attraction is toward their own sex: “*It took time because at the beginning I did not accept this thing […] I do not know, I saw a strange thing, I did not see myself accepted, maybe*” (Interviewee 23, M, 21, urban).

Disorientation characterized feelings such as inadequacy compared to the heterosexist cultural model and disadvantage: “*I thought it would be better if I had never been. But for the situation, for the general discrimination of the thing not because I thought it was a bad thing, just for the easier life I could have. But still, we are what we are*” (Interviewee 19, F, 21, urban).

Therefore, the interviewees claim that they had to go through their own path of personal acceptance, which resulted, in most cases, in a more or less public coming out.

The main difference between the two territorial contexts, therefore, does not concern the process of acceptance and construction of one’s own identity but rather the possibility of revealing one’s sexual orientation to others or not. Different intrapsychic and interpersonal variables have influenced this process. The socio-cultural characteristics of the context they belong to have made finding answers to these questions often painful. In the rural context, young LGB people find it difficult to come out. For example, in some cases, this ended by perpetuating the silence and the need to pretend: “*I cleverly pretended to feel attraction towards the boys, and this led me not to live as many experiences as I wanted*” (Interviewee 6, F, 26, rural).

Participants in an urban context, despite managing to publicly declare their sexual orientation, believe that having to come out is a kind of discrimination, compared to heterosexuals who do not have to do it publicly and whose orientation is taken for granted: “*I have met many people and the question when you meet a homosexual is: are you out? But do your folks know? And so, I learned to answer. […] Still, I’m not saying it so as not to create the superstructure or the hierarchy; it’s something that comes out spontaneously*” (Interviewee 29, M, 29, urban).

Moreover, coming out is problematic in terms of discrimination and harassment for women, who also feel more concern for their physical safety: “*it is doubly difficult for me, because I am a woman and because I am a lesbian and therefore, I have to be careful when I am around with my girlfriend*” (Interviewee 6, F, 26, rural). Therefore, being lesbian is a double minority in a patriarchal context: being women and non-conforming girls.

### The Need for Aggregation and Identification With the Lesbian, Gay, Bisexual Community

The need for aggregation and identification with the LGB community is powerful in both contexts: “*when they are there even just one of these many people, you feel calm so if you have to exchange a kiss or a caress you do it*” (Interviewee 26, M, 25, urban); “*I saw this desire to need to be with someone like me. For example, if a black boy is discriminated against, he goes home and has a black family or at least one of his parents, so there is a necessary belonging, always needed, even more so in those moments. When I returned home, I had a heterosexual family, that is, I had a family, but I was in dire need of people like me*” (Interviewee 10, M, 25, rural).

Common to both contexts, the importance of meeting places for young people emerges; a supportive community contributes to achieving full awareness of oneself: “*it really helps the sense of belonging to someone, of being good for who you are and that’s okay. It helps a lot; it helped me a lot. The sense of belonging was what prompted me to come out*” (Interviewee 12, M, 22, rural).

But a peculiarity of the rural context is the translation of the need to be part of a group into activism which represents the alternative to remaining isolated and invisible.

Indeed, activism represents a strong element of aggregation: “*I started attending Arcigay, slowly I experienced, I met, I shared my story with other people and with the fact that I was equally able to live there without many problems*” (Interviewee 15, M, 25, rural).

### The Role of the Interpersonal Relationship in the Process of Identity Acceptance

This collects all categories referring to family, intimate relationships, and virtual communities.

First of all, the family’s reaction to coming out was not always positive in both contexts. In some cases, people who have suffered violent reactions move away from the family: “*I felt so much anger towards my family, anger that I continue to feel*” (Interviewee 17, F, 21, urban).

Family represents a powerful element of influence since parents are carriers of values and traditions: “*they reproach me for not dressing up as a girl, perhaps even before reproaching me for anything else. They didn’t take my statement well*” (Interviewee 14, F, 24, rural).

But the variables that influence the reactions and the consequent acceptance of their children’s sexual orientation are numerous and closely related to the socio-cultural context.

Participants from the rural context report their parents’ concerns and shame due to the possibility of being judged, criticized, or derided by their fellow citizens: “*Then what will others have to say to me that I have a lesbian daughter?*” (Interviewee 6, M, 26, rural).

And again: “*I cannot accept it, because, in my opinion, it is not like that, it is just your obsession. Don’t you think about the consequences of shame […], the humiliation that we have to suffer, that you have to suffer for a lifetime; at this point, it is better to be alone. If this is to be the case, it is better that you remain alone*” (Interviewee 5, M, 27, rural). This statement also informs us on the beliefs about the origin of homosexuality, which appears to be a whim or something that can be controlled, managed, and, last but not least, inhibited.

For this reason, mainly in the rural context, participants feel the need to move away from their family and therefore from their country of origin: “*the positive rush I had toward my life, so taking possession of my life was the blessing of this home. It was essential for me to have a door that closes and opens as I pleased*” (Interviewee 3, M, 34, rural).

Another participant adds: “*When I moved to a city in northern Italy, I was free, quietly. Because I was far from family ties, however, we are well known in the village, this is also what stops me from unbalancing*” (Interviewee 2, M, 32, rural).

Secondly, intimate relationships mark an essential stage in the process of accepting one’s homosexuality. In this regard, what emerges as data characterizing the experience of young LGB is an almost total lack of stable relationships common to both rural and urban contexts. Specifically, in the rural the small number of inhabitants, (young people in particular) makes it even less possible for young homosexuals to meet a potential partner: “*The context is important; even this affects me a lot, my love life affects me a lot, here there are no possibilities […] Maybe okay, you can have sex, it ends there, that’s not what I’m looking for, which is something solid, someone you can rely on, a relationship*.” (Interviewee 12, M, 22, rural).

In the urban context too, there are relational difficulties but, in this case, they are due to a stereotyped vision of homosexuality: “*it is awful because it seems that homosexuals are more promiscuous than heterosexuals and it is a bad feeling, also because I am not like that, I seek a stable relationship in my life*” (Interviewee 17, F, 21, urban).

The use of the internet represents for both, urban and rural LGB youth the preferred tool to “connect” with the rest of the LGB community, the privileged means of making new acquaintances. All participants believe that virtual reality plays a fundamental role, as they use apps to make love encounters, given the limits of meeting places. For example, in the initial phase of uncertainty about the sexual orientation, they look for same-sex experiences through the web: “*I have always had difficulty in getting involved, let’s say at that sentimental level, and I thought, let’s say it could be a compromise situation, that is, I expose myself up to a certain point, not in real life, but virtual life*” (Interviewee 21, M, 21, urban); or they are looking for possible partners who would otherwise be difficult to meet precisely because of their living in small towns: “*I have difficulty meeting someone because this is the countryside and it is not easy, because it is not possible to leave a cafe and find the man of my life*” (Interviewee 9, M, 25, rural).

### Socio-Cultural Context and Lesbian, Gay, Bisexual Psychological Well-Being

Participants internalize prejudice and social stigma, and in the acceptance phase, their stress level increases exponentially, putting a strain on the psycho-physical well-being of the participants**:** “*I said to myself it would be like this all my life, if that’s already the case at school imagine then at work, when you have a family we don’t talk about it just because I couldn’t think of a family at the time, I felt limited in many things, and it was ugly, so I felt panic*” (Interviewee 20, M, 27, urban) or also: “*I am unhappy for other things. I have many mental problems, in the sense that a person has many hardships in life and my opinion a little related to homosexuality*” (Interviewee 30, M, 30, urban).

In particular, the factor, transversal to both contexts and with a strong impact on the well-being of LGB young people, is internalized homophobia, which represents “*the gay person’s direction of negative social attitudes toward the self*” (Meyer and Dean, 1998, p. 161). In its extreme forms, it can lead to the rejection of one’s sexual orientation. Internalized homophobia is further characterized by an intrapsychic conflict between experiences of same-sex affection or desire to be heterosexual (Herek, 2004). It, therefore, concerns all the factors that reflect the influence that the socio-cultural environment has on the thinking and behavior of young people.

Indeed, participants do not recognize the possibility of naturally exchanging gestures of affection with their partner because they feel the discomfort, feel exposed, and judged: “*I don’t like to do it in the public square where it might bother someone, that is I would feel it offends them if there is one thing that bothers me, I avoid doing it*” (Interviewee3, M, 34, rural), “*At first it was strange for me to walk hand in hand with a man, that is, it bothered me, then over time I got used to it*” (Interviewee 30, M, 30, urban).

A certain degree of non-acceptance of effeminateness is an indicator of internalized homophobia; respondents describe the need to remain within limits and not be too excessive in their behaviors: “*there are too many effeminate people, we are still men, and we must remain so*” (Interviewee 2, M, 32, rural) as well as the need not to “flaunt”: *“without expressing love for a person of the same sex together. If you are in a place where things are accepted, this does not authorize you to exaggerate or externalize this thing. You can live it naturally, but unfortunately, it does not happen; the vast majority of cases, in the places where this thing is accepted, tend to abuse it*” (Interviewee 3, M, 34, rural). It is those who have a negative opinion about homosexual people, who show effeminate behavior in public and accuse them of conducting a bad and deviant image of being homosexual: “*the worst homophobe is gay, I think that becoming a freak phenomenon only makes our situation worse*” (Interviewee 30, M, 30, urban).

Finally, the sentence of a participant about the perception he has of his sexual orientation is significant and reflects the tendency to internalize homophobia: “*it’s not the end of the world, I mean. It is a bit like discovering that you have diabetes; one is not happy with it but lives with it, has a quality of life, an equal life expectancy. Not that I want to assimilate homosexuality to a disease, but surely if I had to express myself, I do not think it is the most beautiful thing in the world, the most normal thing in the world.*” (Interviewee 3, M, 34, rural).

### Ghettoization

*Ghettoization* is the core category that better expresses the condition experienced by the interviewees of Italian rural and urban communities as emerged in the categories defined.

The term “ghetto” refers to areas within European cities that had the aim of keeping the Jewish people “in quarantine” and physically separated from the rest of the population, due to their diverse culture (Žižek, 1997). More generally, Hiebert (2009) defines the ghetto as a place where there is a high residential concentration of a particular social group (such as Jewish, black, gay, etc.), marginalized by the wider society.

Thus “ghettoization” refers to restricting or confining a particular group to a particular social area or category, and implies social isolation (Debnath, 2018).

In our study, Ghettoization emerged as a common psychological condition due to the lack of freedom to express themselves and to increase the awareness of one’s own identity as well as to the sense of isolation caused by the absence of support of the interpersonal network and the wider context.

However, this condition presents different modalities and intensities in the two contexts.

While in the rural area one is alone because of lack of places and people with which to identify and relate to and for the greater social isolation; in the urban area, although there are more opportunities, young people are equally stigmatized and ghettoized because the places they frequent are exclusive to the LGB community. It is, therefore, a group ghettoization rather than an individual one.

In the urban context, the vital need for aggregation emerges, even if there is still the risk of feeling ghettoized in exclusively homosexual places. Participants also affirm that the limit of meeting places is that they only allow sexual relationships, like the online community. At the same time, they would desire to undertake relationships of acquaintance, friendship, and relationships that result in a proper relationship.

**FIGURE 1:**
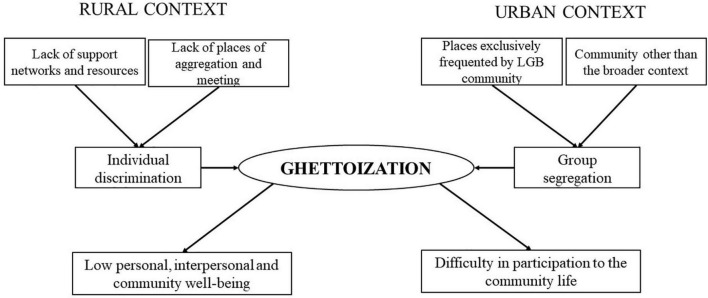
Core category, ghettoization.

Finally, ghettoization seems to have different effects on young LGB people in the two different contexts. While in the rural context it leads to low personal, interpersonal, and community well-being, in the urban context its main consequence is difficulty in participation in the community life (see Figure 1).

This study has highlighted how the life context influences the process of building a solid identity by inducing unstable sentimental relationships.

## Discussion and Conclusion

This study investigated the process of identity acceptance and construction and the well-being of young LGB in rural and urban territorial communities of South Italy, in an ecological approach.

The results lead us to reflect on the close interconnection of individual, relational, and community dimensions, on LGB young people’s well-being (Evans and Prilleltensky, 2007). The participants assume that a socio-cultural element that hinders the process of acceptance of their identities in the territorial communities to which they belong is due to the strong religious tradition that characterizes Italy. In fact, this factor may indeed negatively affect the well-being of LGB young people (Higa et al., 2014). Different studies have shown that the basis of negative attitudes toward gays and lesbians is precisely the system of religious beliefs: many religious denominations deny homosexuality and therefore the faithful internalize these precepts and take a negative view of homosexuality (Whitley, 2009). Therefore, in Italy, and particularly, in Southern Italy, which are bearers of strong religious values and beliefs, the acceptance of homosexuals is more difficult and heteronormativity is more dominant. This can be explained if we consider religion not only as a cultural background but as a real factor capable of shaping a country’s strategies and policies (Fink, 2009).

The relations with the family of origin are undermined by homosexual choice, respect, and social recognition are also harmed. The difficulty of having relationships of respect and recognition within the family, relational and social circle is evident in all the interviews.

Another important result is that for many of the young people interviewed, the university environment, beyond its educational potential, is transformed into a place of opportunities for relationships and encounters (Carbone et al., 2021) where the possibility of experimenting with one’s own identity as gender is facilitated. In this regard, Mitha et al. (2021) research on LGB exclusion experience stated that “*Being geographically distanced from his community, by living away from his family and community, facilitated being able to explore his sexuality without fear or judgment from others*.” (p. 1).

To overcome the difficulties caused by the lack of support received from both the territorial and family context, participation in LGB associations responds to the need for aggregation and identification and coming out of the isolation condition. Therefore, activism offers a chance to break the wall of silence and get out of hiding. Moreover, it will enable action on governments to promote fairer, more connected, and civically engaged societies.

Actively participating in LGBTQ associations is configured as a possibility of improving one’s living conditions and legitimizing one’s identity (Nieves-Lugo et al., 2017).

Participation in feminist or LGBT activities (Watson et al., 2018) and engaging in activism (Singh et al., 2011) is strongly associated with wellbeing. Socio-political involvement or community participation increases well-being by facilitating community connectedness (Prilleltensky, 2005; Gilster, 2012). This is particularly important for young LGB who need social support and identification with their communities to reach an integrated identity and higher levels of well-being. Studies on LGBT youth suggested that community engagement facilitates empowerment, which is related to well-being (Wernick et al., 2014; Wagaman, 2016). Although there is strong evidence that socio-political involvement can lead to well-being in the general population, additional research is needed to understand the relationship between LGBT-specific socio-political involvement for LGBT people.

Some important differences emerged in the two territorial contexts. The rural communities considered are characterized by a lack of support networks and resources for LGB people and the negative climate toward sexual minorities hinders public coming out and, consequently, also hinders the development of support networks among LGB individuals in rural areas (D’Augelli and Hart, 1987). As the research showed, the rural context amplifies in symbolic terms the concern of being discovered, of not being able to create one’s own “space” since there is a lack of places of aggregation and meeting.

Moreover, at a relational level, it remains challenging to develop a relationship free from anxiety, much less if with another homosexual, since he often shares the same fears and anxieties. This situation is reflected in mental states of hopelessness, despair, and self-contempt and in receiving less social and institutional support than urban dwellers (Bell and Valentine, 1995). The ghettoization experienced in rural contexts, therefore, takes on the connotation of social isolation and occurs when the spaces that should provide support to young people (e.g., families, schools, religious organizations, online platforms) create an atmosphere of rejection, bullying, and stigmatization (Garcia et al., 2020) leaving people alone to themselves.

On the other hand, in the urban context of a large town in Southern Italy, although there is a community, someone “*like me*,” it is, in any case, a community outside the community in the broader sense. The difficulty of accessing the broader community generates the perception of ghettoization in the interviewees belonging to urban contexts.

In fact, “LGB neighborhoods,” where people can meet, develop relationships and build communities, social and political spaces to share face-to-face contact (Gieseking, 2016) are created by a homophobic, bi-phobic, and transphobic heterosexual community. Therefore, the areas of homosexual aggregation are experienced as ghettos, in which LGB individuals are confined, and sexuality can be acted out. Still, there are no opportunities to build relationships and bonds. So, these spaces, despite being associated with community and security, are often associated with segregation and stigmatization (Hubbard et al., 2015).

Recently, the new generation of gays and lesbians may find traditional gay villages limiting, considering them as a historical and political area no longer relevant for them (Vaccaro, 2009; Nash, 2013). This is due to greater social and political inclusion, in fact, attitudes about homosexuality have changed and LGBT individuals have become more widely accepted and integrated into mainstream culture (Gratwick et al., 2014) and this allowed young LGBT to be better able to connect with others in different ways which therefore make the gay village superfluous (Usher and Morrison, 2010). In this sense, young LGBT people show a preference for belonging to the community, reducing self-segregation (Hess, 2019).

The ghettoization and the consequent social isolation led to a lack of involvement in the enlarged community with strong negative effects on the well-being of the sexual minority (Higa et al., 2014), also favoring the implementation of risk behaviors for the own health, such as suicide attempts and substance abuse (Garcia et al., 2020).

However, there are some critical limitations. First, this situated research considered two specific contexts of South Italy: a rural area and a large city. Hence, the participants only belong to South Italy and therefore they are not representative of other urban and rural communities in northern and central Italy.

Second, the sampling procedure had to deal with recruiting participants who fully recognize themselves as being gay, lesbian, or bisexual and who wanted to share their visions with the research team. This led specifically to difficulties in recruiting a more significant number of females among the respondents. The number of interviewees is, for the most part, made up of young males, all of the same ethnicity.

This situated study allowed us to understand the different impacts on the well-being of a rural context and an urban one. However, further studies have to develop a better description of these contexts. There is a need to understand LGB well-being in other large cities of different national and international contexts. What is LGB life in a large metropolis such as London and New York or Northern Italy such as Milan? Moreover, what are the relational features of the countryside to consider? Does living in the rural area of Foggia have a similar impact, as, say, in Lancashire County in the United Kingdom?

Finally, the aim of the study was not directed to depict the transgender experience, but this could also be the goal for further investigations.

## Conclusion

In conclusion, our research has highlighted the need to intervene on social, cultural, and relational levels to increase the inclusive capacity of Italian urban and rural territorial communities to promote citizenship rights and equal well-being opportunities for LGB youth.

Well-being for LGB people is closely linked to the possibility of living their choices in a context that protects their rights. In fact, for LGB youth, the interdependence of citizenship and personal rights interfaces with personal and collective well-being.

## Data Availability Statement

The raw data supporting the conclusions of this article will be made available by the authors, without undue reservation.

## Ethics Statement

The studies involving human participants were reviewed and approved by the Ethics Committee of the University of Naples Federico II. The patients/participants provided their written informed consent to participate in this study.

## Author Contributions

BA, AC, and GC contributed to the conception and the design of the study. BA organized the database. IDN and CE performed the qualitative analysis. BA wrote the first draft of the manuscript. BA, CA, and FP wrote the sections of the manuscript. All authors contributed to manuscript revision, read, and approved the submitted version.

## Conflict of Interest

The authors declare that the research was conducted in the absence of any commercial or financial relationships that could be construed as a potential conflict of interest.

## Publisher’s Note

All claims expressed in this article are solely those of the authors and do not necessarily represent those of their affiliated organizations, or those of the publisher, the editors and the reviewers. Any product that may be evaluated in this article, or claim that may be made by its manufacturer, is not guaranteed or endorsed by the publisher.
